# Effect of uniaxial strain on the site occupancy of hydrogen in vanadium from density-functional calculations

**DOI:** 10.1038/srep10301

**Published:** 2015-05-20

**Authors:** Robert Johansson, Rajeev Ahuja, Olle Eriksson, Björgvin Hjörvarsson, Ralph H. Scheicher

**Affiliations:** 1 Department of Physics & Astronomy, Uppsala University, Box 516, SE-751 20 Uppsala, Sweden

## Abstract

We investigate the influence of uniaxial strain on the site occupancy of hydrogen in vanadium, using density functional theory. The site occupancy is found to be strongly influenced by the strain state of the lattice. The results provide the conceptual framework for the atomistic description of the observed hysteresis in the 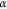 to 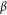 phase transition in bulk, as well as the preferred octahedral occupancy of hydrogen in strained V layers.

Vanadium is a transition metal with electronic configuration 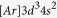 that forms a body-centered cubic structure (bcc). The bcc structure contains tetrahedral and octahedral interstitial sites that can accommodate hydrogen^1^,^2^. In bulk vanadium, hydrogen is found to reside in tetrahedral sites at low concentrations (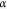-phase), while in the 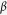-phase H occupies octahedral sites^1^,^2^. The symmetry of the hydrogen induced local strain field is strongly depending on the site occupancy, which is reflected in the hydrogen induced expansion of the lattice^1^,^2^. When hydrogen resides in a tetrahedral site, the local strain field is close to spherical, while it is almost uniaxial when hydrogen resides in the octahedral sites.

In a body-centered tetragonal structure there are three types of tetrahedral sites and three types of octahedral sites, and there are in total three octahedral and six tetrahedral sites per metal atom (Fig. 1). The six tetrahedral sites comprise four 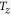 and two 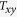 sites (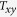 refers to either one of the equivalent 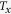 or 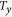 sites). The three octahedral sites comprise one 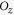 and two 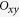 sites. The local elastic response of the lattice arising from the presence of hydrogen in these interstitial sites^3^,^4^,^5^,^6^,^7^,^8^ gives rise to a local strain field, which is the cause of the hydrogen induced expansion^4^. The expansion can be viewed as the sum of the hydrogen induced local strain fields and can therefore, in principle, be used to determine the preferred site occupancy of hydrogen.

The volume changes depend strongly on the boundary conditions and site occupancy, enabling the polarization of the local strain fields. This can, for example, be experimentally accomplished by the use of clamping 9,4 as the preferred site occupancy of hydrogen in vanadium is linked to the strain state of the structure^10^,^11^,^12^,^13^. The hydrogen induced change in volume of a clamped epitaxial film is restricted to the direction perpendicular to the surface. For example, a single crystal vanadium (001) film on a MgO(001) substrate will exhibit a lattice expansion (or contraction) in the (001) direction, independent of site occupancy^10^,^9^. Clamping of V can therefore be used to change the volume expansion, as only 1/3 of the strain field will propagate to the surface when hydrogen is residing in tetrahedral sites. By the same token, the uniaxial component of the local strain field arising from an 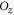 site occupancy will reach the free boundaries and therefore not restrict the expansion. Furthermore, when hydrogen resides in 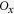 or 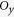 sites, the local strain field cannot give rise to any expansion due to the constraint implemented through the elastic boundary conditions (i.e., clamping) imposed by the substrate. Clamping and straining a V layer will therefore strongly affect the site occupancy, and thereby the observed volume changes^9^,^10^,^13^,^4^.

In the present work we use an atomistic framework and first-principles methodology to investigate the polarization of the local strain fields generated by hydrogen in clamped vanadium and the implications for both site occupancy and lattice expansion. The results allow us to provide a plausible atomistic understanding of the observed hysteresis^2^ in the hydrogen absorption and desorption in bulk V as well as to explain the 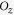 site occupancy at low concentrations in strained V layers^9^. Since in the underlying experiments, the vanadium layers are composed of 21 monolayers^9^, we neglect here any direct interface effects and effectively treat the vanadium host as a bulk material.

## Methods

The calculations were performed using the Vienna *Ab initio* Simulation Package (VASP)^14^,^15^,^16^,^17^. We have used a modified version of VASP in which the National Supercomputer Centre (NSC) at Linköping University had implemented the possibility to perform constrained cell relaxations with one or more lattice vectors fixed, as described in further detail below.

The interactions between the electrons and the nuclei was obtained using the projector-augmented-wave method^18^,^19^. The generalized gradient approximation (GGA) in the parametrization of the Perdew-Burke-Ernzerhof (PBE)^20^,^21^ approach was employed to approximate the exchange and correlation terms in the density functional theory (DFT)^22^,^23^ method. The GGA-PBE method has earlier been shown to be reliable when calculating electronic properties of transition metal hydrides^8^,^9^. A conjugate gradient algorithm was used to relax the atomic nuclei positions to a local minimum in the total energy landscape.

In order to reduce H-H interactions resulting from the imposed periodic boundary conditions while still studying a system that is small enough to be computationally manageable for a large number of calculations, a supercell consisting of 128 vanadium atoms (4 × 4 × 4 bcc unit cells) was constructed to mimic bulk vanadium in which the lowest possible ratio of hydrogen to vanadium [H/V] is 1/128 (corresponding to 0.775 at.% of hydrogen). Due to the rather large dimensions (11.9 Å × 11.9 Å × 11.9 Å) of the supercell, only the 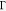 point was used in sampling the Brillouin zone. Comparisons of the total energy, using a 3 × 3 × 3 k-point mesh further established that 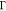 point sampling of the Brillouin zone is sufficient for a quantitative investigation.

Zero-point energy corrections to the total energy are included for the hydrogen atoms and have been calculated from an harmonic approximation of the potential energy change as a function of atomic displacement.

Calculations for higher hydrogen concentrations than [H/V] = 1/128 were carried out by randomly distributing hydrogen atoms into the vanadium supercell and calculating the resulting average volume and energy of 50 structures, with different hydrogen distributions. Hydrogen concentrations [H/V] of 8/128 (5.88 at.%), 16/128 (11.11 at.%), 32/128 (20.00 at.%) and 64/128 (33.33 at.%) were investigated. This corresponds to a disordered state, mimicking the conditions above the phase boundaries of a 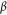-phase.

The 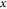 and 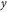 lattice vectors were fixed for all calculations, only allowing lattice relaxation in the 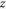-direction. The motivation for this approach is to mimic the conditions of hydrogen uptake in a superlattice where the bottom layer of a thin film of vanadium is held in place through strong bonds to a substrate^9^. This constraint results in a one-dimensional lattice expansion, perpendicular to the plane of the substrate.

It is sufficient to consider the strain in one direction, for example the 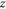-direction, to capture the effect of strain on site occupancy. This implies also that we can treat the 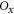 and 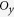 sites to be equivalent when hydrogen is occupying 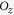 sites in the 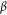-phase. These are identical in the sense that a rotation by 90°around the 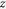-axis will map the 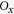 site onto the 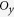 site and vice versa.

## Results & discussion

### Strain and site occupancy

Before presenting the results from our *ab initio* calculations we will provide a conceptual framework for the effect of strain on site occupancy, using a simple hard-spheres model. We will use this approach to estimate the relative energetics of hydrogen occupation in octahedral and tetrahedral sites, solely based on the available interstitial space in a V single crystal. The maximum sphere radius that can be accommodated in the interstitial space formed by metal atom spheres arranged in a bcc lattice is 0.155 for octahedral and 0.291 for tetrahedral sites, in units of the metal atom sphere radius. In the atomistic model used here the vanadium has a Wigner-Seitz radius of 1.217 Å and the corresponding value for hydrogen is 0.370 Å. An octahedral site in V has therefore 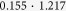
Å = 0.189Å of spherical radius available, which is much smaller than the hydrogen radius. A tetrahedral site provides 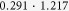 Å = 0.35 Å, which is close to the hydrogen radius. From this consideration, one can see directly that it is not energetically favourable for hydrogen to occupy octahedral sites in an unstrained lattice, because of a large overlap between H and V electrons, raising the total energy through a Born-Mayer repulsion^24^. In the tetrahedral sites, the corresponding density overlap is much smaller, favouring occupation of tetrahedral sites. When the lattice is under uniaxial tensile strain (i.e., 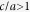) the maximum sphere radius that can be accommodated in the 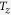 and 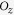 sites is more and more shifted in favour of the 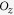 sites. The maximum sphere radius that can be accommodated in 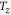 and 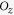 sites becomes equal for *c/a* = 1.118

When the lattice is expanded in the 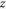-direction the 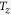 and 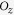 sites are energetically favoured in comparison to their 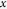
- and 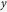-oriented counterparts. This is rather obvious for the octahedral sites but not immediately clear for the tetrahedral sites. For the 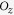 sites, a tensile strain in the 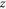-direction will increase the spacing between the vanadium atoms that sit closest to hydrogen, while for 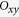 sites, the closest vanadium atoms lie in the 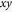-plane, which are geometrically unaffected by the uniaxial strain in the 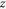-direction.

**Figure 2:**
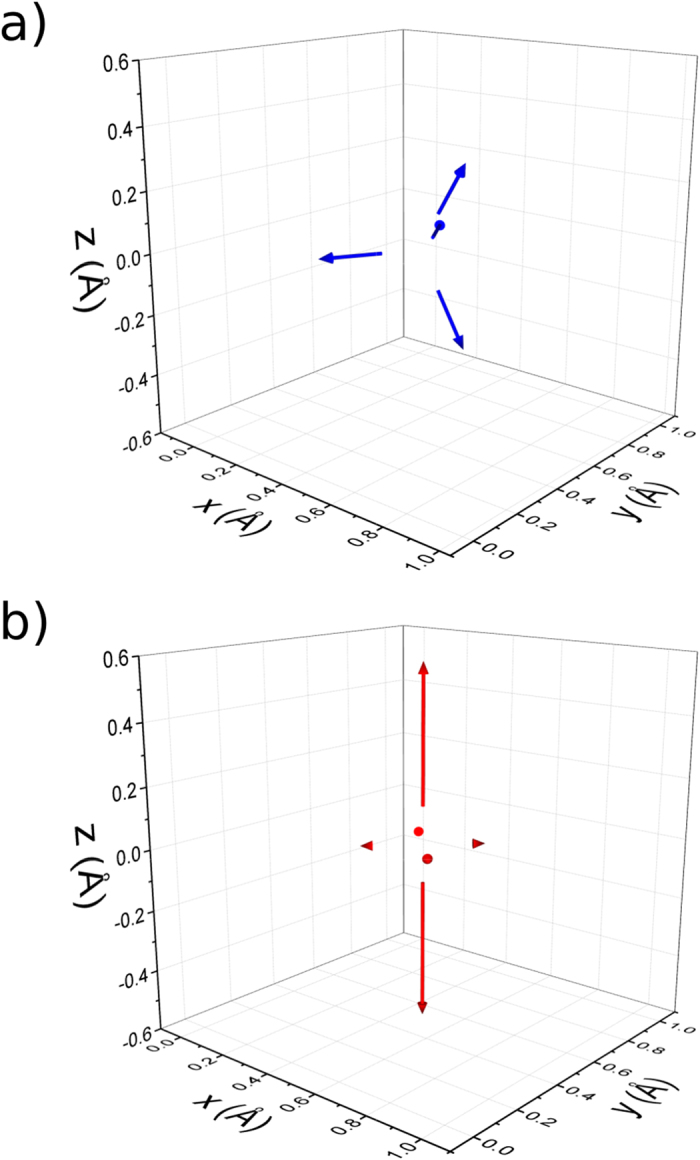
The strain on surrounding vanadium atoms by hydrogen occupying a (**a**) 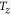 site or (**b**) 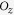 site. The arrows represent the displacement vectors, i.e., by what distance the V atoms have been repelled by the H atom. For clearer visibility, the length of the arrows has been scaled up by a factor of 30.

Figure 2 shows the *ab initio* calculated local strain fields in vanadium caused by hydrogen occupying either a 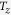 or an 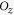 site in a 128 vanadium atoms supercell. The arrows indicate the direction and the magnitude of the displacement of the vanadium atoms. Only the strain on the vanadium atoms in the 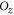 and 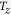 sites are shown (i.e., 4 atoms for a tetrahedral site and 6 atoms for an octahedral site). The isotropic strain field from hydrogen occupying a tetrahedral site and the strongly anisotropic strain field from occupying an octahedral site can be clearly seen in Fig. 2. The “top” and “bottom” vanadium atoms in the octahedron (i.e., the two vanadium atoms that possess the same 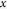 and 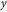 coordinates) are much closer to the hydrogen than the vanadium atoms in the tetrahedron; hence, the former are pushed farther away. In the absence of hydrogen the calculated lattice parameter is 2.99 Å . When hydrogen is placed in the 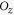 site, the “top” and “bottom” V-atoms are displaced, increasing their mutual distance to 3.35 Å. This local strain corresponds to an increase of 12.1% in spacing between the V atoms which is in excellent agreement with the experimental results of 12.7% for 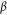-phase VH_0.5_ obtained by EXAFS^10^.

**Figure 3:**
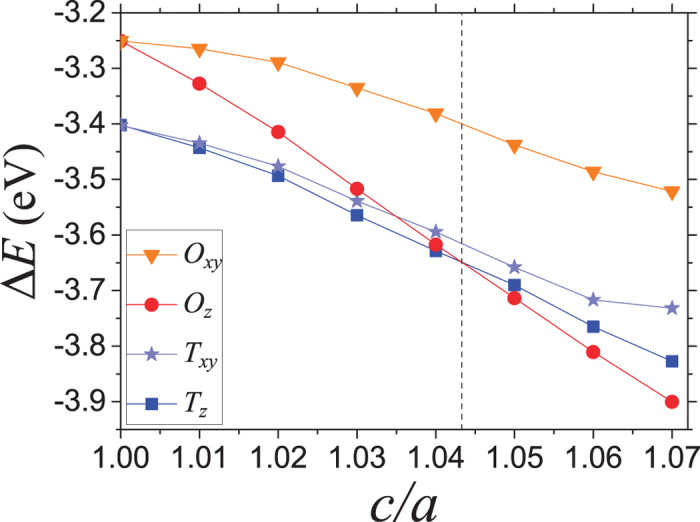
Energy difference as a function of an externally applied global uniaxial lattice strain 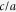 where the difference in total energy 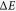 is defined as 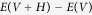. The dashed vertical line at *c/a* = 1.043 marks the critical uniaxial lattice strain for which hydrogen occupancy of 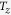 and 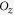 sites becomes energetically equivalent.

To quantify the qualitative ideas obtained from the hard-sphere model, we used *ab initio* total energy calculations to determine the preferred hydrogen occupancy. Fig. 3 shows a plot of the energy for a single hydrogen in a supercell (i.e., a concentration of [H/V] = 1/128, corresponding to 0.775 at.% of hydrogen) occupying a 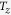, 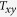, 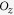 or an 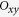 site as a function of the uniaxial strain given in the form of the 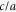 ratio. The vertical axis shows 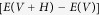 where 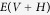 is the total energy of the metal-hydrogen system and 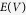 is the total energy of the hydrogen-free vanadium supercell, both calculated at the same 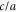 ratio. When the lattice is uniaxially strained (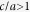), the 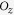 sites will “open up” as described above and become more energetically favoured. This is easily inferred from the results in Fig. 3 since the slope of the 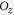 line is larger than that of 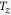, thus at some 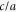 ratio the site occupancy of 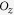 will become lower in energy compared to the 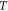 sites. As seen in Fig. 2, the strain is very large in the 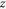-direction for hydrogen occupying an 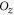 site. A comparison of the strain fields from hydrogen occupation of 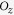 and 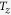 sites shows a larger increase of available spherical radius for the hydrogen occupying an 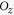 site. This, together with the favourable effect of the uniaxial tensile strain for the occupation of the 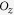 sites makes the 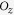 sites energetically favorable already when *c/a* = 1.043. The hard sphere model yielded a transition at *c/a* = 1.118 which can be considered as satisfying agreement when considering the simplicity of the model.

### Concentration dependence of site occupancy

**Figure 4:**
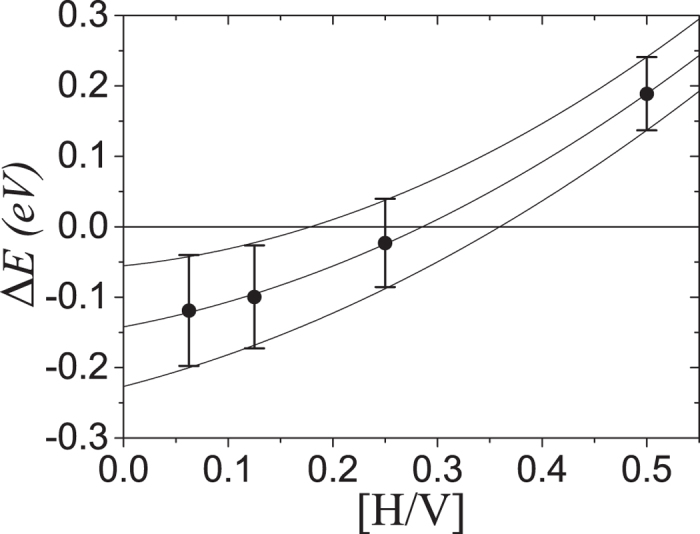
Energy difference between 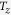 and 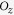 occupancy at optimal 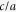 ratios as a function of hydrogen concentration. Here, the energy difference 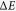 is defined as 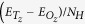 where 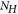 is the number of hydrogen atoms included in the simulation. Data points are for average values and the bars indicate 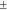 one standard deviation (calculated as the square root of the variance). Connecting lines are second-order polynomial fitting functions.

It is not only the initial strain state which is the source of tetragonal distortion. The hydrogen induced volume changes will also influence the 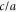 ratio in clamped samples and thereby alter the energy balance between the 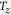 and 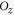 sites. Fig. 4 compares the energies of 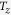 and 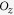 site occupancy at optimal 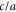 ratios to identify the critical hydrogen concentration where change in site occupancy occurs. The average energy of 50 structures with random hydrogen distributions for four different hydrogen concentrations is calculated (in a disordered phase). Change in site occupancy is approximated to occur between [H/V] of 0.28
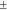
0.07 as this is where the total energy of 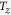 and 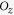 site occupancy becomes equal.

The shift in the site occupancy is found to be driven by energetics rather than entropy. The configurational entropy was determined using Boltzmann’s entropy formula and the internal energy was approximated as the number of hydrogen atoms in 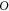-sites times an energy penalty of 0.2 eV (i.e., we approximate that moving a hydrogen atom from a 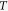 site to an 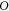 site will raise the energy by 0.2 eV, in accordance with the difference in energy between 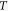 and 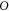 site occupancy, cf. Fig. 3). For all tested hydrogen concentrations and for a broad temperature range, the internal energy is always found to dominate the entropy part, so that coexistence of 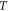 and 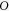 sites is concluded unlikely to occur in the low concentration region. The hydrogen induced lattice expansion can however give rise to change in site occupancy. This can take place in both ordered and disordered phases, thus a 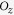 occupancy does not need to imply an ordered 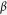-phase and a change of site does therefore not by necessity imply a disorder-order phase transition.

### Volume expansion and hysteresis effects

**Figure 5:**
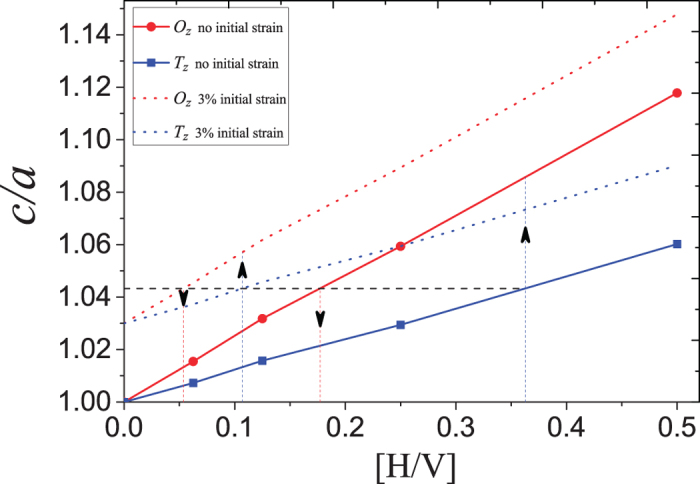
The uniaxial lattice strain 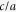 resulting from varying the concentration of hydrogen occupying exclusively either 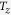 sites (blue data points and lines) or 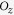 sites (red data points and lines) in clamped vanadium. The horizontal black dashed line at *c/a* = 1.043 marks the critical uniaxial lattice strain for which the hydrogen occupancy of 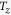 and 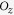 site becomes equal in energy, as seen in Fig. 3. The vertical coloured lines indicate at which hydrogen concentration the critical 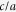 ratio of 1.043 is reached for occupancy of 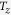 ([H/V] = 0.363) and 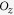 ([H/V] = 0.177) sites, respectively, when there is no initial strain (i.e. *c/a* = 1.00). The dotted lines represent the case of an initial strain of *c/a* = 1.03 before any hydrogen has entered the system. The critical 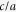 ratio is then reached at [H/V] = 0.107 for 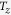 occupancy and 0.054 for 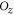 occupancy.

Figure 5 shows the resulting uniaxial lattice expansion (quantified by the 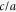 ratio) as a function of hydrogen concentration [H/V]. The relationship between calculated 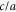 ratio and hydrogen concentration [H/V] is to a very good approximation linear with a slope of 0.120 for 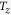 sites and 0.236 for 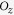 sites, see Fig. 5. These results are in good agreement with the experimental results by Pálsson *et al.*^9^^9^ which determined the expansion to be 0.1189(7) for hydrogen occupation in tetrahedral sites and 0.19(1) for octahedral sites. The calculated change in total volume due to hydrogen occupation of 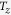 and 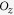 sites are 1.61 Å^3^ and 3.14 Å^3^, respectively, per added hydrogen atom when the expansion is restricted to the 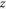-direction (due to clamping). 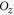 occupancy gives rise to a larger increase in volume, as compared to 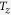 occupancy, due to the anisotropy of the local strain field as seen in Fig. 2. The strain component in the 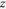-direction is larger for 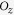 than for 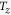 sites, implying that a shift from 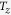 to 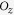 occupancy is accompanied by an increased 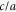 ratio, which favours 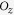 occupancy. The shift in site occupancy from 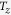 to 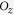 can thus be viewed as a self-amplified process and resembles therefore in many ways a first-order phase transition.

Now we will discuss the difference in the lattice response when the hydrogen concentration is increased or decreased. In an unstrained or nearly unstrained lattice hydrogen is exclusively found in 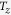 sites. When increasing the hydrogen concentration from low concentrations in clamped V layers, the expansion will open up the 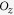 sites, which become energetically favoured above the critical 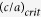 value of 1.043. The uniaxial lattice expansion will therefore result in a shift in site occupancy from 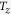 to 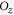 at that concentration. 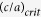 for the change from 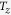 to 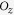 occupancy is marked by a vertical line in Fig. 3 and a horizontal dashed line in Fig. 5.

When starting at a high concentration, all the hydrogen will reside in 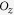 sites. When decreasing the hydrogen concentration, 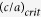 = 1.043 will be reached at [H/V] = 0.177, resulting in a shift in site occupancy from 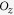 to 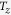. Thus, when increasing the concentration the shift from 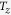 to 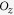 is reached at a different concentration as compared to the change of sites from 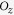 to 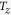 sites when decreasing the hydrogen concentration. Therefore, a hysteresis with respect to lattice expansion is expected when loading and unloading H under the specified conditions and when the thermal excitations are smaller than the energy difference between the two states. These effects do resemble the 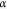 to 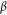 phase transition in bulk V, with respect to both change of sites as well as observed hysteresis^2^. Furthermore, these results clearly illustrate the effect of clamping on the site occupancy, which can be changed without entering the 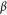-phase in V. When the initial strain of the sample is changed, these boundaries will move as illustrated in Fig. 5: With a biaxial compressive strain in the 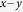 plane, the boundaries will move to lower concentrations and the hysteresis gap will decrease. When 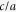 will be larger than a threshold value, hydrogen will solely reside in 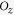 sites, as inferred from experiments^9^

### Summary

The preferred interstitial site occupancy in vanadium with constrained boundaries has been studied using calculations based on density functional theory. The energetics of hydrogen atoms in a bcc-bct supercell were investigated to provide a conceptual understanding of the experimentally observed shifts in site occupancy^9^. In the investigated range of 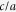 from 1.00 up to 1.07, the tetrahedral (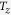) sites are energetically favoured for hydrogen occupation in comparison to the octahedral (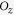) sites in the 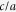 range from 1.00 to 1.043. The octahedral sites are energetically favoured for hydrogen occupation when *c/a* >1.043. The forces exerted on the vanadium lattice by hydrogen atoms occupying interstitial sites will alter the global strain state which in turn triggers a shift in site occupancy above the critical value of *c/a* = 1.043. This self-amplified process can be understood by the obtained strain field from octahedral (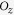) site occupancy which has a larger 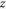-component than that obtained for a tetrahedral (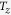) site occupancy. The increase in 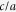 as a function of hydrogen concentration [H/V] is linear and in good agreement with previously obtained experimental results.

The different rate at which the 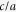 ratio changes as a function of [H/V] for tetrahedral (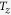) and octahedral (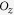) site occupancy has the consequence that the condition for shift in site occupancy is met at different hydrogen concentrations [H/V] when starting from high (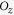) or low concentrations (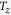). This leads to the theoretical prediction of a hysteresis in the hydrogen loading-unloading process, in which the switch from 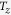 to 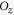 site occupancy and the reverse switch from 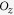 to 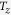 occur at different hydrogen concentrations. The results therefore provide an insight into the interplay between site occupancy and ordering in both bulk and thin films of bcc lattices. The experimentally observed^9^ coexistence of tetrahedral and octahedral hydrogen occupation in the [H/V] concentration range of 0.065–0.068 is an indication that such a hysteresis behavior could indeed be found in vanadium.

## Author Contributions

All authors designed the research, analyzed the data, and reviewed the manuscript; R.J. performed research; R.J., R.H.S. and B.H. wrote the paper.

## Additional Information

**How to cite this article**: Johansson, R. *et al.* Effect of uniaxial strain on the site occupancy of hydrogen in vanadium from density-functional calculations. *Sci. Rep.* **5**, 10301; doi: 10.1038/srep10301 (2015).

## Figures and Tables

**Figure 1:**
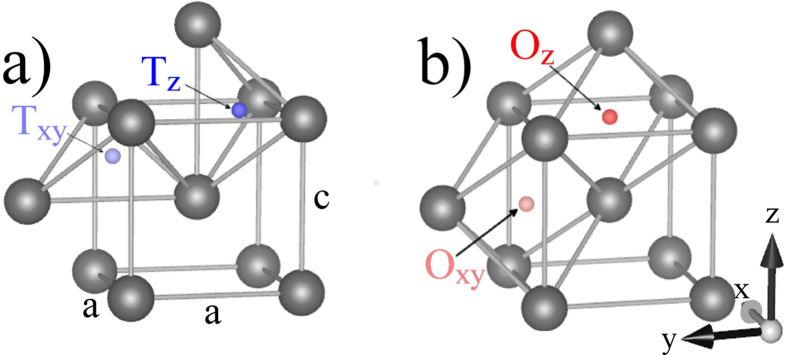
The different types of (**a**) tetrahedral and (**b**) octahedral interstitial sites in bcc vanadium are illustrated here. Large dark spheres represent vanadium atoms and small red, blue, light red, and light blue spheres represent (according to their respective labels) different interstitial positions that hydrogen can occupy. The 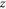-axis is aligned along the vertical direction, while the 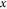
- and 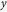-axes lie in the horizontal plane.
